# HIV Futures 8: Protocol for a Repeated Cross-sectional and Longitudinal Survey of People Living with HIV in Australia

**DOI:** 10.3389/fpubh.2017.00050

**Published:** 2017-03-22

**Authors:** Jennifer Power, Graham Brown, Anthony Lyons, Rachel Thorpe, Gary W. Dowsett, Jayne Lucke

**Affiliations:** ^1^The Australian Research Centre in Sex, Health and Society, La Trobe University, Melbourne, VIC, Australia

**Keywords:** HIV, living with HIV, quality of life, antiretroviral treatment, longitudinal design

## Abstract

**Introduction:**

More than 27,000 Australians currently live with HIV. Most of these people have access to quality clinical care and antiretroviral treatment (ART) and can expect good general health. However, HIV-related stigma is a problem and many people living with HIV experience poorer than average mental health. Issues of aging are also of increasing concern. This paper describes the methods and sample for the HIV Futures 8 study, a national survey of people living with HIV in Australia that aimed to identify factors that support health and well-being among this population. HIV Futures 8 forms part of a series of cross-sectional surveys (The “HIV Futures” studies) that have been repeated periodically since 1997. In the most recent survey, participants were able to opt into a prospective longitudinal study.

**Materials and equipment:**

HIV Futures 8 was open to people aged over 17 who were living with HIV. Data were collected in 2015/2016 using a self-complete survey that contained approximately 250 items related to physical and mental health, use of ART, HIV exposure and testing, financial security, social connectedness, relationships, life satisfaction, resilience, stigma, use of health and support services, and health literacy. To enable comparison of cross-sectional data over time, questionnaire items were consistent with those used in previous HIV Futures surveys. In HIV Futures 8, participants were invited to volunteer coded information that will allow longitudinal follow-up when participants complete subsequent HIV Futures surveys. The survey was advertised through the networks of HIV organizations, on social media and through HIV clinics and services. HIV Futures 8 was completed by 895 participants. This represents approximately 3.8% of the total number of people living with diagnosed HIV in Australia in 2014.

**Expected impact of the study on public health:**

Findings from HIV Futures 8 will contribute important insights into the complexity of factors that support physical and mental well-being among people living with HIV. The findings will also assist HIV services to align with broader public health goals related to increasing ART use and improving quality of life among people living with HIV.

## Introduction

The first Australian case of HIV infection was diagnosed in 1982. More than 30 years later, there are now more than 27,000 Australians living with HIV (1). Approximately, 1,000 new cases are diagnosed in Australia each year. This figure increased slightly during the previous decade, from 953 new diagnoses in 2005 to 1,064 in 2012 at which level it has remained relatively stable. There were 1,081 new HIV diagnoses in 2014 (1).

The HIV epidemic in Australia occurs predominantly among gay men and other men who have sex with men (GMSM). Estimates indicate that, in 2014, between 14–18% of Australian GMSM were living with HIV (1). In 2014, 70% of new HIV diagnoses occurred through male-to-male sexual transmission (1). This figure has remained consistent over time (1, 2). Australia is recognized internationally for its early implementation of effective HIV harm minimization and prevention strategies, facilitated through a partnership involving affected communities, government, clinicians, and researchers (3). Compared with other Western countries, the Australian response has successfully minimized HIV cases among injecting drug users, sex workers, and heterosexual populations (3, 4).

However, there is evidence that the Australian epidemic is diversifying (5, 6). In the past 5 years, the rate of new HIV diagnoses among Indigenous Australians has increased, with 16% of these attributable to injecting drug use, a much greater proportion in this transmission category than among non-Indigenous people born in Australia (1). While the proportion of new HIV diagnoses attributable to heterosexual sex has remained consistent nationally for the past decade (at between 18 and 23%) (1), this is not the case in all jurisdictions. In the state of Western Australia, up to 50% of new HIV diagnoses are attributable to heterosexual sex, with 70% of these cases having acquired HIV overseas, most commonly in South East Asia (7). Nationally, there has also been an increase in new HIV diagnoses among people born overseas. In 2014, 39% of new HIV diagnoses attributable to heterosexual sex were among people from high HIV prevalence countries, most commonly low income countries in Sub-Saharan Africa or South East Asia. Among GMSM, the proportion of new diagnoses among men born in Asian countries increased from 6% in 2005 to 15% in 2014 (1). These changing patterns mean the Australian HIV epidemic is now more complex than in the past. Approaches to prevention, testing, and care need to accommodate this changing epidemiology, greater cultural diversity, and global patterns of migration and mobility.

In addition, the nature of HIV prevention is changing largely due to significant advances in biomedical approaches to prevention (8, 9). Highly active combination antiretroviral treatment (ART) became available in Australia in 1997 (10). This was a major advance in HIV treatment that significantly increased health and life expectancy for people living with HIV (11). Since 1997, HIV treatment regimens have become much simpler (in some cases just one pill per day) and have fewer side effects (11, 12). Increasingly, studies are pointing to the long term personal and public health benefits of initiating ART use soon after diagnosis (13, 14). Importantly, the majority of people living with HIV who are on ART are able to maintain viral suppression (15). Some studies have shown that the risk of onward HIV transmission from a person with full viral suppression is close to zero (14, 16, 17). Given the effectiveness of ART at reducing onward HIV transmission, encouraging early and sustained use of ART among people living with HIV has become central to prevention strategies both in Australia and globally (often referred to as “treatment as prevention” or TasP) (18, 19).

In Australia, clinical guidelines regarding prescription of ART were changed in 2015 to facilitate this. Previous guidelines had indicated ART was only appropriate for people whose CD4 count was less than 500 U/ml^3^. Today, there are no such stipulations and early uptake is encouraged (20). International and Australian targets aim for 90% of people living with HIV to be on ART as part of a strategy to reduce, or even eliminate, new HIV transmissions (18, 21). In 2014, it was estimated that 73% of Australians diagnosed with HIV were taking ART and, of these, 92% had achieved viral suppression (1). Some studies have found a slightly higher number are on ART (22). “Treatment as prevention” now sits alongside other interventions including condom use, promotion of HIV testing, and provision of other pharmacological HIV prevention methods including pre- and post-exposure prophylaxis (PrEP and PEP), which are ART medications taken by HIV negative people before or after a potential exposure to HIV to prevent HIV infection (9, 23, 24).

The effectiveness of ART has also generated changes in the age profile of people living with HIV. It has been estimated that by 2020, close to 45% of people living with HIV in Australia will be aged over 50 years (25). Aging in this population has an impact on health and care needs in both the HIV and mainstream health sectors. In particular, there is an increasing need for expertise in the management of health conditions associated with aging (such as heart disease, cancers, diabetes, and neurocognitive impairments) alongside HIV treatment and care (26). The support and social needs of older people living with HIV may also differ from those of younger or newly diagnosed people, meaning the HIV community and support sectors increasingly have to accommodate diverse needs with respect to the age range of their clients and members (27).

Broader cultural and technological changes have influenced the HIV sector and the ways in which people living with HIV seek information and social connection. The online environment now plays an important role in the HIV response as a site of education and information provision (28, 29). It is also a space where people seek out others living with HIV for social connection or support (30) and, increasingly, it is a place where people meet prospective sexual and romantic partners (31, 32). HIV prevention and disclosure of HIV status is managed in unique and evolving ways in online forums, particularly among GMSM.

As a consequence of the changing nature of the HIV epidemic, and of HIV prevention and care, the story of well-being among people living with HIV is shifting and dynamic. Research in this area is still highly relevant, even at a time when HIV treatment is so effective that an HIV diagnosis does not necessarily mean lower life expectancy, poorer health, or poorer quality of life—at least for someone living in a high-income country (33).

However, there are issues that have seen less change over time. Stigma and discrimination associated with HIV are still prevalent (34) and have a negative impact on the lives of many people living with HIV (35, 36). There are ongoing challenges for people living with HIV in the negotiation of intimate relationships, and in relation to disclosure of HIV status to friends, family, and colleagues as well as health-care providers (34, 37). The introduction of TasP and PrEP may also influence the manifestation of stigma. For example, TasP has the capacity to reduce the stigma associated with fear of transmission while simultaneously increasing stigma for those not taking treatment or with detectable viral loads (38). Stigma can affect mental health and well-being at the individual level (35, 39). It can also interrupt and undermine campaigns to increase testing for HIV and other prevention initiatives (40). Stigma is a difficult concept to operationalize or monitor in research as it may manifest in various ways, for example, as outright acts of discrimination, or as a low-level and ongoing anxiety carried by people living with HIV. Often stigma is associated with fear of the possibility of being rejected by, or alienated from, others (37). Stigma also operates at a structural level, implicitly influencing decisions relating to health-care funding, policy direction, or even the criminalization of HIV transmission (40). At all these levels, stigma is a major issue in the lives of people living with HIV that warrants attention in the Australian and international HIV response (34).

### This Study

This paper describes the method and sample of the HIV Futures 8 study. HIV Futures 8 is a cross-sectional survey of adults living with HIV in Australia. The survey forms part of a series of cross-sectional surveys of this population that have been repeated periodically (every 2–3 years) since 1997. HIV Futures 8 is the eighth iteration of this survey. In each survey, data have been collected on issues described above, including aging, stigma, physical and emotional well-being, and ART uptake. When the HIV Futures study was established in 1997, highly active combination ART had only been available in Australia for 12 months. This meant that, while ill-health and HIV treatment remained a major issue of concern for many people living with HIV, other life issues were emerging (or re-emerging) as important—relationships, identity, employment, and housing. Indeed, many people living with HIV were considering returning to work as their health improved with the new treatments. For some people, longer life expectancy required reconsideration of their financial situation (41). As such, the 1997 HIV Futures study was designed to identify and explore needs of people living with HIV in the new era of ART. The study was aimed to be “an investigation of the various ramifications of the changed perception and experience of HIV on the ways that people living with HIV/AIDS lead their lives” (41) (p. 8).

Between July 2015 and June 2016, data were collected for the eighth iteration of the HIV Futures study—HIV Futures 8. To ensure the survey adequately captured the complexities of the current environment with respect to HIV, the survey instrument for HIV Futures 8 was significantly revised from previous versions. This was done through extensive consultation with people living with HIV, HIV community organizations, clinicians, government, and others working in the Australian HIV sector. The updated instrument was designed to capture information relevant to the contemporary situation, while also allowing for comparisons over time with previous versions of HIV Futures. In HIV Futures 8, participants were also invited to opt into a prospective longitudinal study of their health and well-being over time by providing coded information that will enable researchers to match their responses to future HIV Futures surveys.

The aims of the HIV Futures 8 survey were to:

- describe the social and demographic characteristics of people diagnosed with HIV in Australia
- provide a measure of well-being among people living with HIV in Australia that is comparable with previous HIV Futures datasets
- identify factors that support health and well-being among people living with HIV in Australia
- provide data on ART use, and non-use, as well as clinical and community service use among people living with HIV in Australia.

This paper describes the design, instrument, sampling, recruitment, and data collection methods used for HIV Futures 8 and provides an overview of the sample characteristics.

## Materials and Equipment

### Survey Instrument/Measures

HIV Futures 8 is a broad, omnibus survey. The survey instrument contained approximately 250 items as detailed below.

#### Demographic Characteristics

Standard items were used to measure age, sex/gender, place of residence (postcode), religion, highest education level, total household income, current employment, current relationship status, gender and sexuality of partners over lifetime, Australian residency, and visa status.

#### Financial and Housing Security

Standard items were used to ask participants about their current housing arrangements, including the people with whom they live and type of housing (rental, owned, public, others). One item asked participants if they had experienced difficulty managing the cost of basic items for living (utility payments, rent, food, and so forth) in the past 12 months. This was a modified version of an item used in the Australian Household Income and Labour Dynamics Australia (HILDA) survey, a national population-based survey of Australian households. Two other items from the HILDA survey were also included: capacity to raise $3,000 in a short period of time and source of this revenue (42, 43).

#### HIV Exposure and Testing

Participants were asked: which year they tested positive for HIV; the year they believe they acquired HIV; the means by which they acquired HIV (sex with a man, sex with a woman, injecting drug use, blood products, other); the location in which they acquired HIV (Australia/overseas). Participants were also asked the results of their most recent CD4 count and viral load testing. These items all align with previous HIV Futures questionnaires, allowing comparison between each survey from 1997 onward. In HIV Futures 8, items were also added about whether participants had ever used PEP or PrEP medication prior to testing positive for HIV and whether they used contract tracing/partner notification services after diagnosis.

#### ART Use and Adherence

Participants were asked whether they currently use ART and which ART medications they currently use. Respondents not currently using ART were asked whether they had used ART in the past and to indicate their reasons for not taking ART.

To measure attitudes toward ART use, participants were asked to respond to six statements relating to beliefs and attitudes about beginning ART and its safety and effectiveness. Responses were recorded on a 4-point Likert scale, “strongly disagree” to “strongly agree.” These questions were all comparable to previous versions of HIV Futures.

One item asked participants to indicate what percentage of time they adhered to their ART regimen in the past month (44). A further item asked participants to identify the main reasons why they missed doses of ART in the previous month, and this is comparable to the AIDS clinical trial group adherence baseline questionnaire (45). Several items asked participants to report on any problems they have with ART use including experience of side-effects, whether they had recently changed treatment combination, the reason for this change, difficulties accessing prescriptions, and cost of filling prescriptions. These questions were all comparable to previous versions of HIV Futures.

#### Health-Related Quality of Life

Several items together can be used to build a concept of overall health-related quality of life including general physical and mental well-being, life satisfaction, social connectedness, and resilience. These measures are all listed below.

#### Physical and Mental Health

The survey included the RAND Short Form 36 1.0 (SF 36) (46) that has been validated for use with people living with HIV (47). This is a widely used measure of health-related quality of life that incorporates physical and mental health as well as functional and role impairment. Functional impairment is also measured using one item, “Do you regularly need help with daily tasks because of long-term illness or disability?” which is derived from the Australian Longitudinal Study of Women’s Health (48).

Participants were also asked whether they had ever been diagnosed with a mental health condition or a range of other physical health conditions, including hepatitis B or C. One item asked participants to identify any sexually transmissible infection they had been diagnosed with in the past 12 months. One item asked participants if they had been affected by symptoms of advanced HIV disease (AIDS-related illnesses) in the past 12 months. A further general item asked participants to rate their overall sense of well-being using a 4-point Likert scale. This general item was also included in previous HIV Futures surveys.

#### Satisfaction with Life

One item with several sub-questions asked participants to indicate their satisfaction with life in a range of areas including employment, housing, safety, community, health, neighborhood, and leisure time. This was followed by a global measure of overall sense of life satisfaction. Both items used a 0–10 scale, with 0 indicating completely dissatisfied and 10 indicating completely satisfied. These items were also designed to be comparable with the HILDA survey (49).

#### Resilience

HIV Futures 8 included the 10-item version of the Connor-Davidson resilience scale (50), in which participants were asked to respond to a series of statements relating to resilience and coping. Participants scored each item on a 5-point Likert scale, ranging from 0 (not true at all) to 4 (true nearly all the time). Scores were summed to a maximum of 40. A higher score indicates higher resilience (51).

#### Social Connectedness and Support, Including with Other People Living with HIV

General sense of connectedness to others was measured using 10 items in which participants were asked to signal the extent to which they agree with 10 statements relating to friendship and support. Scores were recorded on a 7-point Likert scale. These items were derived from the HILDA survey (52). Following Baker (52), scores were calculated so that they ranged from −30 to 30 with a score closer to −30 indicating that the person perceives they have very little support or friendship available to them.

Participants were asked to indicate whether they personally know any other people who are HIV positive, how much of their free time is spent with other HIV positive people and whether they have contact with HIV-related organizations. These items were consistent with previous HIV Futures questionnaires.

A series of questions were also included that related to sense of connectedness to the lesbian, gay, bisexual, and transgender (LGBT) community. These were adapted from Frost and Meyer’s LGBT community connectedness scale (53). These questions were only asked of men who identified as gay or bisexual.

#### Stigma and Discrimination

The instrument included the Berger HIV Stigma Scale (54), a 40-item scale that measures different constructs related to HIV stigma to produce four subscales: (1) personalized stigma, which relates to the impact of others knowing about the participant’s HIV status, including loss of friends or avoidance of others; (2) disclosure concerns, which measures difficulties related to disclosure or hiding HIV status from others; (3) negative self-image, which measures shame, guilt, and negative self-worth associated with HIV status; and (4) public attitudes, which measures participants’ perceptions of what others think about people living with HIV. Participants responded to each item using a 4-point Likert scale. Responses were scored from 1 to 4 giving total possible scores of 40–160, with higher scores indicating greater stigma.

#### Use of Health Care Services

Participants were asked whether they have a Medicare card (Australian national health insurance, which is not available to non-residents) and whether they have private health insurance. Participants were also asked: which health-care professional/s they see for general and HIV-specific care and treatment; frequency of medical visits; use of home-based medical care; frequency of medical visits, costs associated with medical care; and distance traveled to access HIV specialist care. These questions were all comparable to previous HIV Futures surveys.

#### Health Literacy

The questionnaire included 12 items comprising three subscales that measured: feeling understood and supported by health-care providers; ability to engage actively with health-care providers; capacity to navigate the health-care system. These subscales were part of the Ophelia Understanding Health and Health-care Questionnaire (55).

#### Alcohol and Other Drug Use

The questionnaire included two items measuring use and frequency of tobacco smoking. The items were derived from the national Drug Strategy Household survey, 2013 (56). Three items that comprise the AUDIT-C questionnaire were included to measure frequency and quantity of alcohol consumption (57).

Frequency and impact of other drug use was measured using the following items: frequency of non-prescribed drug use in the past 12 months; the extent to which non-prescription drug use interferes with daily life; whether participants had been diagnosed with a substance misuse disorder in the past 12 months; and, if yes, whether treatment had been received for this. Questions relating to use of non-prescription drugs were consistent with previous HIV Futures surveys.

#### Relationships and Safe Sex

Participants were asked whether they had a current romantic partner or partners, the HIV status of those partners, satisfaction with their sexual and emotional relationships with their partners, and use of safe-sex methods within their current relationships, including condoms and their partner’s use of pre- or post-exposure prophylaxis. These questions were all comparable to previous HIV Futures surveys. Participants were also asked about sexual partners over the past 6 months including regular and casual partners. With respect to participants’ most recent sexual encounters with casual partners (where applicable), participants were asked whether they were aware of their partners’ HIV status and their own disclosure of HIV status and safe-sex practices on those occasions. For GMSM, questions about casual sexual relationships in the past 6 months were designed to be comparable with the Gay Community Periodic Survey, a survey of gay and other homosexually active men (both HIV positive and negative) conducted in Australia every 2 years (58).

#### Criminalization

There were six items that asked direct questions about the extent to which participants were aware of Australian laws regarding requirements for disclosure of their HIV status to sexual partners and the impact of such laws on their decision-making regarding disclosure and condom use.

#### Attitudes toward Participation in Research toward a Cure for HIV

A series of items were included to explore the attitudes of Australians living with HIV toward recent clinical research aimed toward developing a cure for HIV. Participants were asked to rank, from 1 to 6, the possible benefits of a HIV cure. The options included social benefits, such as not being considered a person with HIV, and medical benefits such as no longer being able to transmit HIV to others. These scenarios were adapted, with permission, from a previous Australian survey of clinical trial patients run by McMahon and colleagues (59) who had derived them originally from the European community survey on HIV cure (60). To identify characteristics of participants who would be willing to participate in a trial, the survey instrument included one primary outcome measure: If you had the opportunity to participate in an HIV cure-related clinical trial beginning tomorrow, how willing would you be to participate? Responses were recorded on a four-point Likert scale (1: not at all willing to; 4: very willing). Where participants indicted they were willing to participate in a HIV cure trial, a series of questions was asked of them relating to possible social and personal benefits. These questions were adapted, with permission, from Arnold and colleagues’ US-based survey of people living with HIV (61).

## Stepwise Procedures

### Cross-sectional Study Design and Sample Size

HIV Futures 8 is a cross-sectional survey of people living with HIV in Australia, which forms part of a series of repeated cross-sectional surveys of this population that began in 1997. Data were collected between July 2015 and June 2016 using a self-complete instrument that could be filled in online or using a hardcopy booklet that was supplied to prospective participants with a reply paid envelope. The survey instrument contained approximately 250 items as described above.

Since 1997, HIV Futures surveys have consistently achieved sample sizes of between 850 and 1,200 participants (22, 62–65). A similar sample size was the target for HIV Futures 8. This sample size has proven necessary in analysis of previous HIV surveys to ensure that subgroups, particularly those based on age and gender, are large enough to ensure appropriate reliability (66, 67).

### Longitudinal Design and Sample Size

In HIV Futures 8, participants were invited to provide information to generate a unique participant code. This code enabled their responses to HIV Futures 8 to be paired with their responses to future versions of the survey—data collection for HIV Futures 9 is due to commence in 2018. Information used to generate the participant code included month and year of birth, first letter of first name, first letter of middle name (if applicable), and first letter of surname. The code therefore allows pairing between surveys to establish longitudinal data while also ensuring participants remained anonymous. Participants were not asked to supply contact details within the questionnaire; however, those who completed the questionnaire online were able to open a separate online form in which they could leave their contact details to receive information about subsequent HIV Futures surveys. Participants who completed a hardcopy version of the questionnaire could email their contact details for inclusion on the contact database. All people on the contact database will be informed about HIV Futures 9 in 2018 and any future surveys.

Preliminary analysis of HIV Futures 8 data indicates that over 750 participants provided information to generate a unique participant code to enable longitudinal follow-up of their responses. We are aiming for a sample of 350 of these people in HIV Futures 9 in 2018. This sample size is large enough to allow for robust statistical analysis and sub-group analysis, but is based on an assessment, determined by previous comparable research conducted by members of the research team (67), that approximately 50% attrition is likely.

### Recruitment

HIV Futures 8 was open to people living with HIV, aged over 17 years and currently living in Australia. Given there is no available sampling frame, this study relied on a self-select sample. Participants were recruited through electronic advertising in a range of forums including advertisements sent through the email lists of HIV community organizations; advertising on relevant websites; social media advertising, particularly Facebook advertisements targeting GMSM as well as general Facebook users who had interactions with HIV-related events such as World AIDS Day; advertisements on “dating apps” used by GMSM; and flyers and posters displayed in HIV clinics. Social media strategies also included peer leaders promoting the survey to their networks through established private “Facebook” groups for people living with HIV. These networks were also used to provide regular updates on recruitment progress to the online community. Hard copies of the survey were distributed through the mailing lists of HIV community organizations and made available in the waiting rooms of HIV clinics and community services.

### Data Analysis

Data analysis will involve descriptive analysis of items related to ART use, ART adherence, physical and mental health, alcohol and other drug use, relationship status and safe-sex practices, and use of clinical and support services. Logistic and hierarchical regression modeling will be used to identify predictors of well-being. Outcome measures in these analyses will include physical and mental health, resilience, social connectedness, and perceptions of stigma. Analyses will also be run to identify predictors of well-being in specific subgroups including women, people aged over 50, people aged under 35, and people diagnosed with HIV in the previous 5 years. Where population data are available for particular measures, such as the SF 36, comparisons will be made with published findings. Data from HIV Futures 8 will be compared with previous HIV Futures datasets to track changes over time in: self-reported health and well-being, treatment use, attitudes toward treatment use, and feelings about disclosure of HIV status. Changes over time will be measured using basic statistical techniques (*t*-tests, analysis of variance test, and/or chi-square tests) to examine differences between each consecutive year or between an identified baseline year and other years. The baseline may be the first iteration of HIV Futures in 1997, or another iteration of the survey as relevant (for example, it may be more relevant just to examine changes over the past 10 years). A series of short topic-specific broadsheet reports will be generated and distributed to stakeholders and community as they become available to ensure rapid distribution of results and to sustain engagement in the study over time. For this paper, data were analyzed descriptively to describe socio-demographic characteristics of the sample.

### Ethics

Ethics approval for this study was granted by the La Trobe University College of Science, Health and Engineering Human Ethics Committee (SHE CHESC S15-100) as well as two community-based ethics committees: ACON (formerly the AIDS Council of NSW) and the Victorian AIDS Council. The key ethical concerns with this study relate to the importance of protecting confidentiality of participants through secure data storage and avoiding publication of any potentially identifiable information. Information about HIV and counseling services and support options were made available to all participants who completed the study.

## Anticipated Results

Over the past 20 years, HIV has shifted from being considered an untreatable, potentially fatal illness to a manageable chronic condition in Australia where people generally have ready access to ART. The HIV Futures studies chart the impact of this change on issues related to everyday living with HIV, such as financial security, employment, social and intimate relationships, stigma and discrimination, general well-being, and, increasingly, aging. HIV Futures 8 will build on the existing dataset, while also exploring issues related to the contemporary experience of living with HIV. In particular, these data will provide information about attitudes toward ART use among people living with HIV at a time when the policy and clinical focus on treatment as prevention has been rapidly evolving. HIV Futures 8 will also yield important findings on the experience of aging with HIV. Since 1997, the average age of HIV Futures participants has increased significantly. In HIV Futures 8, we anticipate there will be capacity—due to a larger number of participants aged over 50—to explore the impact of aging on the management of comorbidities, social connectedness, and financial security.

### Participants

HIV Futures 8 was completed by 895 people living with HIV in Australia. This represents approximately 3.8% of the number of people living with diagnosed HIV in Australia in 2014 (1).

The majority of respondents completed the survey on-line (65%, *n* = 582), while 35% (*n* = 316) completed a hard copy of the survey. There were 338 participants (38%) who indicated they had participated in a previous HIV Futures study, while 363 (41%) indicated HIV Futures 8 was the first HIV Futures survey they had completed (the remainder of participants were unsure if they had completed a previous survey or they did not respond to the question). To gauge effectiveness of recruitment techniques, participants were asked where they found out about the study. The most common response was an email or (hardcopy) mail out from an HIV organization (*n* = 283, 32%), followed by an email that was sent to people who were on a contact list from previous HIV Futures surveys (*n* = 202, 23%). Social media advertising also proved successful with 127 (14%) seeing a “Facebook” advertisement, 53 (6%) seeing a post to a Facebook group for people living with HIV, and 37 (4%) seeing an advertisement on a phone “app”. There were 127 (14%) who picked up a hardcopy of the survey from a clinic or HIV organization.

The socio-demographic characteristics of the sample are shown in Table 1. These characteristics broadly reflect the pattern of the HIV epidemic in Australia with respect to gender and sexuality (1). The majority of participants were men (91%, *n* = 804), over 80% (*n* = 747), identified as gay or bisexual men, and the most common mode through which participants acquired HIV was male-to-male sex (see Table 2). The average age of participants was 51 years and over half the sample (54.2%, *n* = 485) were aged 50 years or older. There has been a steady increase in the average age of participants since the first HIV Futures survey in 1997, when the average age was 39 years (41). In 2012 (HIV Futures 7), the average age was 49 years (22).

**Table 1:** **Characteristics of participants**.

	*N* (%) Total *n* = 895
**Age [mean (range)]**	51 years (range 19–86 years)
**Gender**
Men	804 (90.5)
Women	74 (8.3)
Transgender	6 (<1)
Other or non-specified gender (excludes missing = 7)	4 (<1)
**Sexuality**	
Gay men	697 (78.7)
Bisexual men	50 (5.6)
Heterosexual men	38 (4.3)
Lesbian/bisexual women	6 (<1)
Heterosexual women	65 (7.3)
Other (excludes missing = 10)	29 (3.2)
**Aboriginal or Torres Strait Islander**	21 (2.3)
**Country of birth**	
Australia	649 (72.5)
UK	68 (7.6)
New Zealand	42 (4.7)
European countries	36 (4.0)
Asian countries	29 (3.2)
African countries	17 (1.9)
USA and Canada	11 (1)
South America	9 (<1)
Middle east	5 (<1)
Pacific	3 (<1)
Unspecified	26 (2.9)
**Language**	
English spoken at home	854 (97.7)
English is first language	792 (88.5)
**Residency**	
Australian citizen or permanent resident	842 (97.6)
**Work status**	
Working full time	341 (38.6)
Working part time	134 (15.2)
Home duties/unemployed not seeking work	64 (7.2)
Unemployed and seeking work	52 (5.9)
Retired/not working	160 (18.1)
Student	38 (4.3)
Disability support	47 (5.3)
Other (casual work, volunteer, carer) (excludes missing = 11)	48 (5.4)
**Household income (Australian dollars)**	
$0–$49,999	377 (42.6)
$50,000–$99,999	258 (29.2)
$100,000–$149,999	102 (11.5)
$150,000+	89 (10.1)
Don’t know or prefer not to say (excludes missing = 11)	58 (6.6)
**Highest level of education**	
Less than year 12 (high school certificate)	130 (14.7)
Year 12 certificate	94 (10.6)
Tertiary diploma or trade certificate	265 (29.9)
University degree (excludes missing = 8)	398 (44.8)
**Place of residence**	
Inner city/inner suburban	532 (60.7)
Outer suburban	109 (12.4)
Regional center (population 5,000+)	151 (17.2)
Rural (excludes missing = 18)	85 (9.7)
**Relationship status**	
Cohabiting with partner/spouse	276 (30.9)
Single (not in a relationship)	504 (56.3)
In a regular relationship with one or more partners	332 (37.1)
Other or unspecified	59 (6.6)
**Children**	
Have children (total)	177 (19.8)
Have children (men)	125 (15.5% of men)
Have children (women)	48 (64.8% of women)
Have children (trans or non-specified gender)	3 (30% of trans/ns gender)
Currently living with dependent children	30 (3.4)
**Number of years since HIV diagnosis [mean (range)]**	15.2 years (range 1–35 years)

**Table 2:** **Comparison of HIV Futures 8 sample with Annual Surveillance Report 2015 and the AHOD Annual Report 2016**.

	HIV Futures 8 (%)^a^	Surveillance Report (%)^b^	AHOD (%)^c^
**Gender**			
Men	91	91	92
Women	8	Not reported separately	8
Transgender	<1	Not reported separately	0
**Aboriginal or Torres Strait Islander**	2.3	1.8	2
**Exposure category**			
Male-to-male sex	80	67	72
Male homosexual contact and Injecting drug user (IDU)	<1	4	3
IDU	2	3	2
Heterosexual sex	7	13	17
Receipt of blood/blood products	<1	1	1
Other	4	13	2
Missing	6	–	2
**Born outside Australia**	28	40	18
**Taking ART**	97	73	97

*^a^Percentages exclude missing data, N = 895*.

*^b^Gender and exposure categories include all new HIV diagnoses in Australia up to 2014 (*N* = 35,122), estimates of Aboriginal and Torres Strait Islander people and people born outside Australia based on estimates of people living with HIV in 2014 (*N* = 27,150)*.

*^c^Characteristics based on all patients enrolled in Australian HIV Observational Database (AHOD), *N* = 4,270, percentage of people currently taking antiretroviral treatment (ART) based on patients under active follow-up in 2015, *N* = 2,408 [Source: Ref. (1, 68, 69)]*.

This was a well-educated sample, with 45% holding a university degree. However, over 40% had a household income of less than AUD$50,000 per annum, well below the average Australian household income in 2014 (70). The majority of the sample was born in Australia (*n* = 649, 73%) and spoke English as their first language (*n* = 792, 89%). This is likely to reflect an under representation of people from non-English-speaking migrant communities, which will be discussed with respect to study limitations below.

We have compared key demographic characteristics of the HIV Futures 8 sample with published reports of sample characteristics from two other Australian datasets: The Australian HIV Observational Database (AHOD) Annual Report 2016, and the HIV, Viral Hepatitis and Sexually Transmissible Infections in Australia Annual Surveillance Report 2015 (henceforth referred to as the Annual Surveillance Report) (see Table 2). AHOD is a prospective cohort study of HIV infected individuals attending specialized general medical practitioner sites, sexual health clinics, and tertiary referral centers throughout Australia (68). The study began in 1999 and currently has 4,270 patients enrolled, with 2,408 under active follow-up in 2015 (69). The Annual Surveillance Report incorporates information from multiple sources including the National HIV Registry, which is a record of each new HIV diagnoses in Australia, and the Australian Pharmaceutical Benefits Scheme, which records information on prescriptions obtained for ART (1). Table 2 shows key characteristics of the sample for each dataset. In all three samples, over 90% of participants were men. HIV Futures 8 had a slightly higher percentage of participants who had acquired HIV *via* male-to-male sex (80% compared with 67% for the Annual Surveillance Report and 72% for AHOD). This may be because there is a strong connection between Australian LGBT communities and the HIV community organizations that assisted in promoting HIV Futures 8. Consequently, gay and bisexual men may have been more likely to know about the HIV Futures 8 study than heterosexual men. Both the AHOD and HIV Futures 8 samples had similar figures with respect to ART use (97 and 96%, respectively). By comparison, the Annual Surveillance Report shows an estimated 73% of people diagnosed with HIV using ART in 2014. Both AHOD and HIV Futures 8 capture data from people who are more likely to be connected to clinical services and so more likely to be using ART. Figures in the Annual Surveillance Report take into account people who are newly diagnosed with HIV who may not yet have commenced ART or be established in clinical care. Among HIV Futures 8 participants, people who had been living with HIV for 5 years or less were significantly less likely to be on ART than those who had been living with HIV for 6 years or more, χ^2^ (1, *N* = 857) = 16.11, *p* < 0.001.

## Discussion

HIV Futures is the largest and longest-running study of the health and well-being of Australians living with HIV. This is the major strength of this study. It is a unique dataset that collects information on a comprehensive array of demographic attributes, social circumstances, life experiences, and health-related issues among people living with HIV. These data can be used to build a nuanced understanding of the complexity of factors that support a good quality of life among people living with HIV. The study is based on recognition that living with HIV can have a significant impact on an individuals’ well-being even when their physical health is well supported by ready access to ART and quality clinical care. These data contribute to a growing body of international data on the health, well-being, and quality of life of people living with HIV—including the relationship between quality of life and the clinical management of HIV in Western countries. For example, data from HIV Futures 8 include measures that can be used to explore similar themes to those that have been a focus of analyses of the UK-based “Antiretroviral, Sexual Transmission Risk and Attitudes” dataset (71–74).

The socio-demographic characteristics of the HIV Futures 8 sample broadly match that of the Australian population of people living with HIV based on a comparison with other national datasets. The validity of self-select samples in epidemiological and health-based research is subject to debate (75). When studies are advertised widely through channels such as social media, and participants opt into the study, it is impossible to determine a response rate or compare responders with non-responders to assess representativeness of the sample (75). However, there are some noted strengths of self-select samples recruited online, such as a greater capacity to attract “invisible” or hard-to-reach populations (76, 77). Some studies indicate that creative approaches to recruitment *via* social media can achieve representative samples of particular groups (77). A lack of comparable, population-wide datasets makes it difficult to know whether participants in the HIV Futures 8 survey are representative of the population of Australians living with HIV. Comparisons with the Annual Surveillance Report and the AHOD data suggest that people from non-English-speaking migrant communities are under represented in this survey, which we will discuss further in the limitations section below. The HIV Futures 8 sample is also likely to include a higher proportion of people currently using ART than the overall population of people living with HIV in Australia, because HIV Futures 8 is advertised through HIV clinical and support services. People who are yet to be engaged with treatment and care are less likely to have been exposed to advertising about the study.

The HIV Futures 8 sample size of 895 was slightly smaller than has been achieved for previous HIV Futures surveys. Both HIV Futures 6 in 2008 and HIV Futures 7 in 2012 had a sample of over 1,000 participants (22, 65). Recruitment for HIV Futures 8 was largely centered on social media and online advertising. While this was effective in promoting the study, HIV Futures 8 was competing for attention with other online surveys targeting people living with HIV that were recruiting at the same time—multiple surveys being, in part, a function of the cost-effectiveness and ease of conducting online surveys. This may have created a sense of “survey fatigue” among people living with HIV. It is also possible that being HIV positive is less central to people’s lives than it was 10 or 15 years ago due to improved treatment and physical well-being. For this reason, people may feel less compelled to take part in HIV-related well-being studies than they were in the past.

Engagement with the HIV community sector was central to successful recruitment in this study. Extensive consultation with individuals working in the HIV sector was undertaken to ensure the design and content of HIV Futures 8 was relevant and useful for services providers and community agencies. This ensured the study had endorsement and support from the community sector. As well as being ethically appropriate, this facilitated recruitment as HIV community organizations were able to promote the study to their members—a strategy that was highly effective.

### Limitations

There are some limitations to this study that should be noted. The survey instrument for HIV Futures 8 was long and required a reasonably high level of English literacy to complete. This meant it would have been difficult for some people to complete the survey without assistance from a translator or support person. As a way to make the survey more accessible for people with lower English and/or literacy skills, we simplified the language in the opening section of the survey instrument (Part 1) as much as possible, while still ensuring it included core questions about health and well-being. We informed participants that it would be adequate for them to complete only Part 1 of the survey if it was challenging for them to continue. The Flesch Reading Score for Part 1 was 74%, indicating it should be readable for people with approximately seventh grade English literacy skills. In the Flesch system, a score of 90–100 is considered approximately fifth grade reading level, while a score of 0–30 indicates college or university graduate reading level. Limited resources meant we did not have capacity to undertake the more intensive recruitment that would be required to engage properly with many non-English-speaking migrant populations in Australia. In many migrant communities in Western countries, including Australia, HIV is very a hidden issue and there are high levels of stigma (78, 79). The process of building trust and rapport with community leaders and community members at a level necessary to encourage participation in a study related to HIV would be intensive and take an extended period of time. We acknowledge that this is important work, however, that we will endeavor to develop in an alternative project in this area.

A limitation of the sampling method is that it relied heavily on advertising through social and support organizations for people living with HIV. While we made efforts to promote the survey through broader channels, particularly social media, people not connected to such organizations may have been less likely to be aware of the study has potential to introduce some sampling bias. Furthermore, while social media advertising may have been effective in targeting people less connected with the HIV service sector, those who are isolated from both services and social media channels (particularly those relating to HIV and/or LGBT communities) may have been less likely to be exposed to survey advertising.

## Ethics Statement

This study was carried out in accordance with the recommendations of Australian National Health and Medical Research Council National Statement on Ethical Conduct in Human Research, La Trobe University College of Science, Health and Engineering Human Ethics Committee (SHE CHESC S15-100) with written informed consent from all subjects. All subjects gave written informed consent in accordance with the Declaration of Helsinki. The protocol was approved by the La Trobe University College of Science, Health and Engineering Human Ethics Committee (SHE CHESC S15-100).

## Author Contributions

All authors contributed to the study design, and development of the survey instrument and recruitment. JP and RT conducted data analysis. JP prepared the first draft of the manuscript, and all authors contributed to further developing and editing the manuscript.

## Conflict of Interest Statement

The authors declare that the research was conducted in the absence of any commercial or financial relationships that could be construed as a potential conflict of interest.
